# Independent Vector Analysis for Feature Extraction in Motor Imagery Classification

**DOI:** 10.3390/s24165428

**Published:** 2024-08-22

**Authors:** Caroline Pires Alavez Moraes, Lucas Heck dos Santos, Denis Gustavo Fantinato, Aline Neves, Tülay Adali

**Affiliations:** 1 Center for Engineering, Modeling and Applied Social Sciences (CECS), Federal University of ABC (UFABC), Santo André 09280-560, SP, Brazil; heck.l@ufabc.edu.br (L.H.d.S.); aline.neves@ufabc.edu.br (A.N.); 2 Department of Computer Engineering and Automation (DCA), Universidade Estadual de Campinas (UNICAMP), Campinas 13083-852, SP, Brazil; denisf@unicamp.br; 3 Department of Computer Science and Electrical Engineering, University of Maryland, Baltimore County (UMBC), Baltimore, MD 21250, USA; adali@umbc.edu

**Keywords:** brain–computer interface, electroencephalogram, motor imagery, independent vector analysis, autoregressive model

## Abstract

Independent vector analysis (IVA) can be viewed as an extension of independent component analysis (ICA) to multiple datasets. It exploits the statistical dependency between different datasets through mutual information. In the context of motor imagery classification based on electroencephalogram (EEG) signals for the brain–computer interface (BCI), several methods have been proposed to extract features efficiently, mainly based on common spatial patterns, filter banks, and deep learning. However, most methods use only one dataset at a time, which may not be sufficient for dealing with a multi-source retrieving problem in certain scenarios. From this perspective, this paper proposes an original approach for feature extraction through multiple datasets based on IVA to improve the classification of EEG-based motor imagery movements. The IVA components were used as features to classify imagined movements using consolidated classifiers (support vector machines and K-nearest neighbors) and deep classifiers (EEGNet and EEGInception). The results show an interesting performance concerning the clustering of MI-based BCI patients, and the proposed method reached an average accuracy of 86.7%.

## 1. Introduction

The brain–computer interface (BCI) enables a direct connection between the brain and the external world. Electroencephalography is a technique capable of translating brain activities into commands based on scalp-recorded measurements [1,2]. Some benefits of using this BCI method are its non-invasive nature, safety, high temporal resolution, and relatively low cost. All these advantages have attracted the interest of the scientific community, and as a result, electroencephalogram (EEG) signals have been used in the study of several areas, such as epilepsy and brain tumor detection [3], alternative communication channels for disabled patients [4], emotion recognition [5], and neuromuscular disorders [6]. Among all EEG-based applications, the motor imagery (MI) paradigm is probably one of the most popular. It refers to the imagination or mental rehearsal of a motor movement without any real motor execution [7,8].

The MI paradigm has been employed in cognitive psychology and cognitive neuroscience to explore the unconscious structure that anticipates a movement execution. In some contexts, such as medical, athletic, and musical areas [9,10,11], a mental rehearsal can be as effective as an authentic physical proceeding, which leads to a promising future therapeutic tool to improve the performance of motor functions in patients with damage to the central nervous system [9].

There are several proposed methods to identify the MI movements from EEG signals as accurately as possible, most of them focused on feature selection or classification algorithms. However, feature extraction over EEG signals for BCI systems has shown to be a crucial stage for classification performance.

BCI Competition III Dataset 4a (DS4a) is a widely recognized motor imagery dataset that has been extensively studied, leading to the development of various techniques. For instance, Na Lu et al. [12] introduced a method known as structure-constrained semi-nonnegative matrix factorization (NMF), which extracts key EEG patterns in the time domain by enforcing mean envelopes of event-related potentials (ERPs) as constraints. This approach, called SCS-NMF, achieved an accuracy of 68.94%. On the other hand, Rasool Ameri et al. [13] developed a dictionary pair learning (DPL) method for EEG classification, using L0- and L1-norm calculation to obtain sparse coefficients via linear projection, resulting in an accuracy of around 80%. More recently, researchers in [14] proposed a framework combining bispectrum, entropy, and common spatial pattern (BECSP) for feature extraction from MI-EEG signals, achieving an accuracy of 84.91% after selecting the most interesting ones, through a tree-based method. Additionally, the study in [15] compared three popular signal decomposition techniques—empirical mode decomposition, discrete wavelet transform, and wavelet packet decomposition—for EEG classification, with wavelet packet decomposition (WPD) sub-bands yielding an average accuracy of 92.8% for DS4a. Despite these consistent results, DS4a classification remains a challenge. Considering the previous methods, the possibility of working with only one subject may not be enough to deal with a multi-source retrieving problem in some cases. Assuming that the motor imagery data are collected from several subjects executing the same task, the problem can be extended to a multi-model approach, such as independent vector analysis (IVA), which could explore the dependences across subjects.

IVA was firstly proposed for the separation of convolutive mixtures in the frequency domain [16], considering the joint blind source separation (JBSS) problem [17]. Since then, there have been applications in fMRI (functional magnetic resonance imaging) [18,19] from HD-sEMG (high-density surface electromyography) [20], multimodal neuroimaging data fusion [21], and EEG data as muscle artifact removal [22]. However, the IVA application in MI-based BCI as a feature extraction technique is a pioneering approach. This work proposes a novel perspective of MI classification through a new feature extraction method: a combination of IVA and Autoregressive (AR) models. The method is applied to the motor imagery dataset from the BCI Competition III Dataset 4a. Classification is obtained through different methods: support vector machines (SVM), K-nearest neighbors (KNN), EEGNet, and EEG-Inception, in order to evaluate the efficiency of the obtained features. Comparing the results with the ones in the literature, this novel approach showed a homogeneous accuracy performance.

In Section 2, we describe the JBSS problem and the IVA method. Section 3 shows the AR model and the classifiers applied in this work. Section 4 presents BCI competition III dataset 4a and the data preprocessing. The simulation results are presented and analyzed in Section 5. Finally, we conclude this paper in Section 6.

## 2. Joint Blind Source Separation

In certain applications, such as neurodiagnostic applications [23], dealing with multiple datasets is a necessity, which leads to multisubject/multimodal data fusion. In these cases, the task of blind source separation may be extended to JBSS, which exploits correlations across datasets (inter-set dependence) while still searching to recover independent latent sources within a dataset (intra-set independence) [19]. The general concept of the JBSS problem involves *K* datasets, each containing *M* independent sources and *N* samples. Such a mixing process can be modeled by the following equation:

(1) $$x^{\left[k\right]}\left(n\right)=A^{\left[k\right]}s^{\left[k\right]}\left(n\right),\;1\leq n\leq N,\;1\leq k\leq K,$$

where $s^{\left[k\right]}\left(n\right)={\left[s_{1}^{\left[k\right]}\left(n\right),\ldots ,s_{M}^{\left[k\right]}\left(n\right)\right]}^{T}\in R^{M}$ is the concatenated source vector of the *k*-th dataset, ${\left(\cdot \right)}^{T}$ denotes the vector transpose, $A^{\left[k\right]}\in R^{M\times M}$ is the *k*-th invertible mixing matrix, both assumed unknown, and $x^{\left[k\right]}\left(n\right)={\left[x_{1}^{\left[k\right]}\left(n\right),\ldots ,x_{M}^{\left[k\right]}\left(n\right)\right]}^{T}\in R^{M}$ is the concatenated mixture vector of the *k*-th dataset.

Following the recommendation of [17], it is important to perform data whitening prior to performing source separation. Considering $V^{\left[k\right]}$ as the whitening matrix of the *k*-th dataset, $z^{\left[k\right]}\left(n\right)=V^{\left[k\right]}x^{\left[k\right]}\left(n\right)$ is the whitened mixture signal.

The demixing process aims to find matrices $W^{\left[k\right]}$ and the corresponding source vector estimates $y^{\left[k\right]}\left(n\right)$ for each one of the *K* datasets. Hence, the separation system is given by

(2) $$y^{\left[k\right]}\left(n\right)=W^{\left[k\right]}z^{\left[k\right]}\left(n\right),\;1\leq n\leq N,\;1\leq k\leq K.$$

The mixing matrices are potentially distinct for each dataset and are not necessarily related, admitting permutation and/or scale ambiguity.

### 2.1. Independent Vector Analysis

Independent vector analysis is a powerful approach for solving the JBSS problem, and is an extension of independent component analysis (ICA) to multiple datasets by leveraging the dependence across datasets [16,17]. In [19], the role diversity, i.e., different statistical properties, is explained for both ICA and IVA, and the application of both to medical image analysis is discussed. When applied to multiple datasets, Group ICA (GICA) is a widely used approach [24,25]; however, it is noted that it has limitations when compared to IVA in terms of common group-level spatial maps and inter-subject variability preservation [26,27]. The statistical dependence modeled through a multivariate probability density model provides full interaction across the datasets, making IVA an attractive method for problems such as subgroup identification when used with multisubject data [19]. Ref. [28] showed encouraging results of IVA application to subgroup identification, revealing significant differences between the identified subgroups. Such studies demonstrate the ability of IVA to deal with subject variability and subgroup identification, highlighting the advantages of IVA in dealing with multiple datasets.

In IVA, the components from a particular dataset are assumed to be statistically independent of each other, as in ICA methods. However, in contrast to ICA, IVA also exploits the dependence between correlated components from different datasets. These correlated components are regrouped into the so-called source component vectors (SCVs). The *m*th SCV can be written as $y_{m}={\left[y_{m}^{\left[1\right]},\ldots ,y_{m}^{\left[K\right]}\right]}^{T}\in R^{K}$, which is statistically independent of all the other SCVs [17]. The IVA cost function is given by

(3) $$I_{IVA}=\sum_{m=1}^{M}H\left[y_{m}\right]-\sum_{k=1}^{K}\log\;\left|\det\left(W^{\left[k\right]}\right)\right|-C_{1},$$

where H[·] is the entropy function, $\det(W^{\left[k\right]})$ is the determinant of matrix $W^{\left[k\right]}$ and $C_{1}$ is a constant term that depends only on $x^{\left[k\right]}$.

The mutual information part of the IVA cost function is responsible for solving the permutation ambiguity that occurs in the JBSS problem [17,19]. Furthermore, the minimization of the cost function (3) simultaneously minimizes the entropy of all components and maximizes the mutual information within each estimated SCV [17]. In addition, IVA has shown, in most cases, good performance in capturing variability in spatial components across datasets [25,27]. In this paper, we work with IVA-G [17] that only takes second-order statistical information into account, assuming multivariate Gaussian distributions for the SCVs.

### 2.2. IVA Using Vector Gradient Descent

In [17], the authors describe four algorithms for minimizing the IVA cost function given by (3). Within these alternatives, the vector gradient descent algorithm was chosen in this paper due to the decoupling method that enables the tailoring of the step size for each direction, resulting in faster convergence per iteration than that achieved in traditional methods [29]. Using this approach, the IVA cost function (3) is differentiated with respect to $w_{m}^{\left[k\right]}$ [17], where $w_{m}^{\left[k\right]}$ is the *m*th row of $W^{\left[k\right]}$:

(4) $$\begin{array}{cc} \frac{\partial I_{IVA}}{\partial w^{\left[k\right]}} & =E\left\{\phi^{\left[k\right]}\left(y_{m}\right)z^{\left[k\right]}\right\}-\frac{h_{m}^{\left[k\right]}}{{\left(h_{m}^{\left[k\right]}\right)}^{T}w_{m}^{\left[k\right]}}, \end{array}$$

where $\phi^{\left[k\right]}\left(y\right)=\frac{\partial \log p(y_{m})}{\partial y_{m}^{\left[k\right]}}$, $p(y_{m})$ is the pdf (probability density function) of $y_{m}$, and $h_{m}^{\left[k\right]}$ can be defined as a unit length vector such that ${\tilde{W}}_{m}^{\left[k\right]}h_{m}^{\left[k\right]}=0$, where ${\tilde{W}}_{m}^{\left[k\right]}$ is the (M−1)×M matrix obtained by removing the *m*th row of the demixing matrix $W^{\left[k\right]}$ [17,29].

The gradient obtained by (4) is used to iteratively adapt each demixing row of $W^{\left[k\right]}$:

(5) $${\left(w_{m}^{\left[k\right]}\right)}_{it+1}\leftarrow {\left(w_{m}^{\left[k\right]}\right)}_{it}-\mu \frac{\partial I_{IVA}}{\partial w_{m}^{\left[k\right]}},$$

followed by a normalization step:

(6) $${\left(w_{m}^{\left[k\right]}\right)}_{it+1}\leftarrow \frac{{\left(w_{m}^{\left[k\right]}\right)}_{it+1}}{∥{\left(w_{m}^{\left[k\right]}\right)}_{it+1}∥},$$

where μ is the adaptation step size and it represents each iteration.

## 3. Classification Algorithms

Having extracted the features through IVA, a dimensional reduction is highly recommended before classification. Thus, an autoregressive (AR) model is used [30,31], and its weights are extracted for the classification step. This step will be detailed in the sequel, followed by a brief description of the classification methods SVM, KNN, EEGNet, and EEG-Inception.

### 3.1. Autoregressive Model

The AR model is frequently used to represent a random process in view of preserving their important attributes and also reducing the data dimension. This is possible due to the model structure, where the output variable linearly depends on its own previous values [32]. Thus, an autoregressive model of order *q* describes the signal *u* as follows:

(7) $$u\left(n\right)=a_{1}u\left(n-1\right)+a_{2}u\left(n-2\right)+\cdots +a_{q}u\left(n-q\right)+\nu \left(n\right),$$

where ν(n) is a white noise with zero mean and variance $\sigma_{\nu }^{2}$, and {$a_{1},\ldots ,a_{q}$} are the AR parameters that can also be written as $a=[a_{1},\ldots ,a_{q}]$.

Based on the Yule–Walker equations [33], the coefficients of the AR model can be estimated by

(8) $$\hat{a}=R_{u}^{-1}r_{u},$$

where $R_{u}=E\left[u\left(n-1\right)u^{T}\left(n-1\right)\right]$, $r_{u}=E\left[u\left(n-1\right)u\left(n-q+1\right)\right]$ and $u\left(n-1\right)={\left[u\left(n-1\right),u\left(n-2\right),\ldots ,u\left(n-q\right)\right]}^{T}$. In this paper, an AR model is obtained for each estimated source ($y_{m}^{\left[k\right]}$) in each *k*-th dataset.

### 3.2. Classifiers

SVM is an efficient supervised algorithm based on statistical learning theory that can be used for classification or regression problems [34]. While in other methods, the separation hyperplane normally assumes distributed class-conditioned data, SVM seeks to find the separation hyperplane with the largest margin between classes.

The KNN classifier is one of the most popular neighborhood classifiers in pattern recognition [35]. It is a nonparametric supervised learning classifier that uses the majority within the K-closest training examples to classify or predict an individual data point. The previous described algorithms are well-known in the machine learning field.

Nevertheless, deep learning approaches have been attracting the attention of the scientific community, and have presented promising results in biomedical engineering applications. EEGNet [36] is a compact convolutional neural network for EEG-based BCIs. The method uses depthwise and separable convolutions to construct an EEG-specific network that encapsulates several well-known EEG feature extraction concepts, such as optimal spatial filtering and filterbank construction, while simultaneously reducing the number of trainable parameters when compared to other networks. More recently, a deep learning model has been proposed by E. Santamaria-Vazquez et al. [37], called EEG-Inception. This method integrates the inception modules for event-related potential (ERP) detection, which can be efficiently combined with other structures in light architecture and requires very few calibration trials.

## 4. Experimental Setup

In the previous section, we described a method for feature extraction based on IVA. In order to better understand the proposed method, in this section, we investigate the performance of the algorithm by applying it to a real EEG dataset for motor imagery movements. In the following, we describe this dataset and the preprocessing stages.

### 4.1. Dataset Description—BCI Competition III Dataset 4a

Dataset 4a from BCI Competition III (DS4a) is provided by B. Blankertz et al. [38], and contains data recorded from 5 subjects—identified as “aa”, “al”, “av”, “aw”, and “ay”—using 118 channels sampled at 1000 Hz, which were downsampled to 100 Hz. The cue-based BCI paradigm involves two motor imagery tasks: imagining right-hand movement and right-foot movement, totaling 280 trials per subject.

During each trial, a fixation cross was shown to each subject, followed by a short acoustic warning tone, indicating the beginning of the trial. Two seconds later, a cue arrow pointing either right or down appeared on the screen for 3.5 s, instructing the subjects to perform the corresponding motor imagery task. Subjects were to continue the motor imagery task until the arrow disappeared, after which a brief black screen signaled a short break.

### 4.2. Proposed Method

In order to evaluate the proposed method, initially, we split the dataset into training and test data using k-fold cross-validation with $k_{f}$ = 10. However, since the IVA matrix initialization (e.g., based on a Gaussian distribution) is a relevant stage for feature extraction and to maintain the test data unknown, using the training data, we also considered a hold-out sample technique of 10% as a validation dataset to investigate the IVA initialization effect on the performance of the method. In addition, each time series given by the EEG signal was separated into window samples of 4 s according to each motor imagery class.

#### 4.2.1. Training Stage

**Figure 1:**
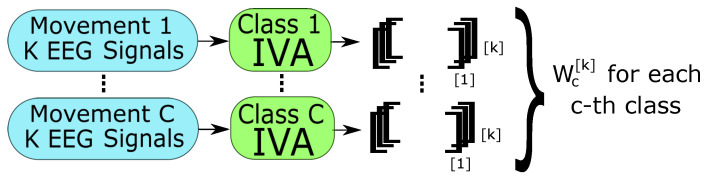
Procedure description to obtain the IVA matrices $W_{c}^{\left[k\right]}$ for each class based on the training data.

**Figure 2:**
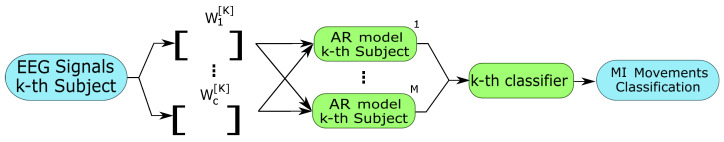
Procedure description for the *k*-th subject used in training and test datasets.

Firstly, in the training stage, the data were whitened, as recommended in [17], for each subject, separately. The EEG signal collected from each subject was considered to be one dataset. IVA was applied in the training data for each class separately to obtain the $W_{c}^{\left[k\right]}$ matrices that correspond to the extraction of the main features for the *c*-th class and *k*-th subject, with k∈{1,…,5} for DS4a, and c=1,2. This procedure is presented in Figure 1. Then, considering the *k*-th subject, the estimated SCV components were obtained by multiplying the training and validation data by each class matrix $W_{1}^{\left[k\right]}$ and $W_{2}^{\left[k\right]}$, followed by each corresponding whitening matrix $V_{1}^{\left[k\right]}$ and $V_{2}^{\left[k\right]}$, resulting in $y_{c_{train}}^{\left[k\right]}$ and $y_{c_{valid}}^{\left[k\right]}$, as shown in Algorithm 1. Using both matrices at this point is necessary considering that validation and test data are assumed to be completely unknown. The choice of obtaining IVA matrices for each class can leverage the feature extraction process, leading to a possible classification improvement. Subsequently, $y_{1}^{\left[k\right]}$ and $y_{2}^{\left[k\right]}$ were stacked and AR modeling was applied to each extracted feature (corresponding to each EEG channel) in order to reduce and adjust the data dimension. The resulting AR parameters were used as the classifier inputs. Finally, the data of each subject were classified according to the two considered classes. This second step is exemplified in Figure 2.

Optimization of the IVA cost function, given by (3), is not an easy task. As usually occurs with gradient descent-based algorithms, initialization plays a crucial role. In order to better explore the method’s potential, a search for a good $W_{c}^{\left[k\right]}$ initialization was implemented using the validation data. The suitable initialization for $W_{c}^{\left[k\right]}$ is denoted as $W_{selected_{c}}$.

To select the appropriate $W_{selected_{c}}$, SVM and KNN classifiers were chosen (block diagram of Figure 2), since both are low-cost, well-established algorithms and can provide a feasible direction to find the suitable $W_{selected_{c}}$. More details will be discussed in Section 5.1. When using deep learning approaches, given by EEGNet and EEG-Inception, the AR parameter extraction step is not necessary. Thus, $W_{selected_{c}}$ and the training data were used directly.

#### 4.2.2. Test Stage

After selecting the suitable initialization, $W_{selected_{c}}$, the procedure described in Figure 2 is reapplied using the test data, where the estimated SCV components are represented by $y_{c_{test}}^{\left[k\right]}$. The whole method is summarized in Algorithm 1. The IVA and classifier weights obtained in the training stage are kept constant and applied to the test data. This procedure was used to classify the motor imagery movements between the right hand (RH) and right foot (RF) for DS4a.

In the following, methods will be named after the classifier used: IVAS for SVM, IVAK for KNN, IVAEN for EEGNet, and IVAEI for EEG-Inception.
**Algorithm 1** IVAS, IVAK, IVAE or IVAEI
Initialization parameter algorithm: *q*, μ - **Training Stage:** **for** each initialization $W_{c}^{\left[k\right]}$ **do**   $W_{c}^{\left[k\right]}\leftarrow$ random initialization;   **for** each class *c* **do**     **Apply IVA** - input: $z_{train}^{\left[k\right]}$; output: $W_{c}^{\left[k\right]},$k∈{1,…,5} and c=1,2   **end for**   **for** each subject *k* **do**     **for** each class *c* **do**          $y_{c_{train}}^{\left[k\right]}=\left[\begin{array}{c} W_{1}^{\left[k\right]}V_{1}^{\left[k\right]}x_{c_{train}}^{\left[k\right]}\\ W_{2}^{\left[k\right]}V_{2}^{\left[k\right]}x_{c_{train}}^{\left[k\right]} \end{array}\right]$ and $y_{c_{valid}}^{\left[k\right]}=\left[\begin{array}{c} W_{1}^{\left[k\right]}V_{1}^{\left[k\right]}x_{c_{valid}}^{\left[k\right]}\\ W_{2}^{\left[k\right]}V_{2}^{\left[k\right]}x_{c_{valid}}^{\left[k\right]} \end{array}\right]$       **end for**     $y_{train}^{\left[k\right]}\leftarrow \left[y_{1_{train}}^{\left[k\right]}\;\;y_{2_{train}}^{\left[k\right]}\right]$ and $y_{valid}^{\left[k\right]}\leftarrow \left[y_{1_{valid}}^{\left[k\right]}\;\;y_{2_{valid}}^{\left[k\right]}\right]$       **AR model** is applied for each channel and subject, according to Equation (7)     **SVM and KNN** classifier-training with $y_{train}^{\left[k\right]}$, evaluated with $y_{valid}^{\left[k\right]}$-output-movement classification accuracy   **end for** **end for**  - $W_{c}^{\left[k\right]}$ with the highest accuracy - ${W_{\mathrm{selected}}}_{c}^{\left[k\right]}$ for each subject and class    
- **Test Stage:** **for** each subject *k* **do**     $y_{test}^{\left[k\right]}=\left[\begin{array}{c} {W_{\mathrm{selected}}}_{1}^{\left[k\right]}V_{1}^{\left[k\right]}x_{test}^{\left[k\right]}\\ {W_{\mathrm{selected}}}_{2}^{\left[k\right]}V_{2}^{\left[k\right]}x_{test}^{\left[k\right]} \end{array}\right]$       **if SVM or KNN then**     input: Extract AR parameters from $y_{test}^{\left[k\right]}$; output: MI classification   **end if**   **if EEGNet or EEGInception then**     input: Apply directly $y_{test}^{\left[k\right]}$; output: MI classification   **end if** **end for**


## 5. Results and Discussion

In order to evaluate the algorithm’s performance, in this section, we analyze the effect of IVA initialization, the number of EEG channels considered, and correlation cross-subjects for dataset DS4a. To analyze such aspects, we fixed IVA adaptation step size to μ=1 and the number of AR coefficients q=4, based on our previous work in [39].

### 5.1. IVA Initialization

**Figure 3:**
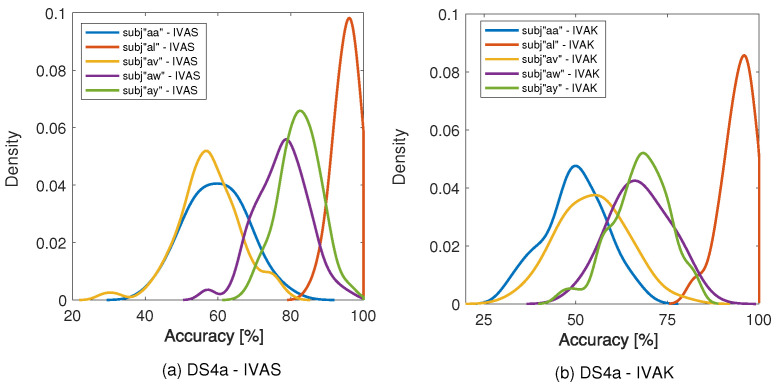
Performance analysis of IVAS and IVAK concerning IVA initialization based on the KDE for subjects from Dataset4a. (**a**) Dataset4a with IVAS; (**b**) Dataset4a with IVAK.

In Section 4.2, we described the IVA matrix selection methodology, which is grounded on a random IVA initialization search. Concerning the number of initialization iterations, for the sake of computational efficiency and based on pre-analysis, 100 iterations were used to select the appropriate $W_{selected_{c}}$. In that sense, IVA was randomly initialized 100 times using a Gaussian distribution with zero mean and unit variance, and for each initialization, accuracy was computed based on the parameters of the classifiers and validation dataset, and considering the same IVA matrix initialization for all subjects. Figure 3 shows the kernel density estimation (KDE) for DS4a and two algorithms: IVAS and IVAK. In both cases, it is possible to verify the occurrence of an initialization that maximizes accuracy, even if it may be a rare event. In Figure 3a, the subjects “aw” and “ay” show a longer tail and similar pattern, finding initializations with accuracies over 90%, and the subject “al” has the highest accuracy probability. On the other hand, in Figure 3b, for instance, three out of five subjects present a greater likelihood for accuracy around 80%, while the curves obtained for subjects “aa” and “av” present a mean achieved accuracy lower than the one obtained by the other subjects, showing a probable higher classification complexity.

Based on these analyses of the initialization that maximizes accuracy, the matrix that leads to the greatest accuracy is chosen for the dataset and subjects (measured in the validation set), and applied to the test dataset.

### 5.2. Number of EEG Channels

**Figure 4:**
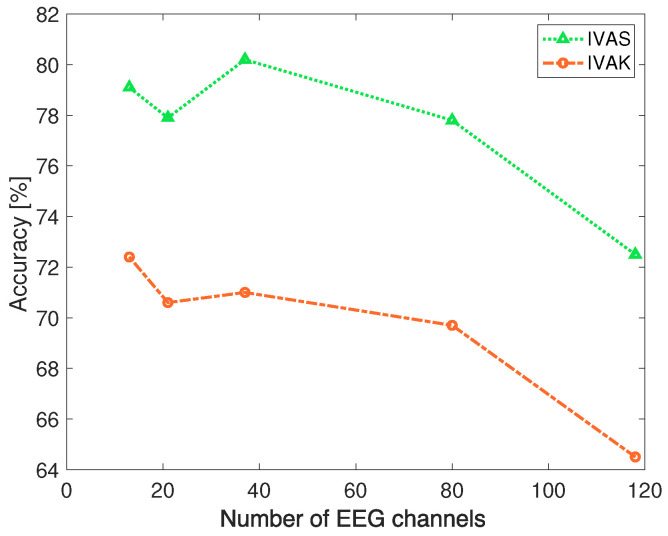
IVAS and IVAK performance analysis with respect to the number of EEG channels.

Another interesting analysis is to investigate the algorithm’s performance with respect to the number of EEG channels used as each IVA dataset input. Considering that DS4a EEG signals have 118 channels, the number of channels was analyzed using 13, 21, 37, 80, and 118. To reduce the number of channels, those located in brain regions known for higher activity during motor imagery tasks were chosen [40]. The results are shown in Figure 4. The results show that using all the available channels leads to a decrease in performance. The best results for DS4a were obtained by IVAS, with 37 EEG channels (FAF5, FAF1, FAF2, FAF6, F7, F5, F3, F1, Fz, F2, F4, F6, F8, FFC7, FFC5, FFC1, FFC2, FFC4, FFC6, FFC8, FT9, FT7, FC3, FC1, FCz, FC2, FC4, FC6, FT8, FT10, CFC7, CFC5, CFC3, CFC1, CFC2, CFC4, CFC6). Ideally, as the number of channels increases, more information is available. However, we hypothesize that the amount of noise also increases and could prejudice the feature extraction process. Among the algorithms tested, SVM was the one that performed better. In this case, the IVAK algorithm presented the lowest performance when the number of EEG channels was the maximum 118 channels.

### 5.3. Correlation Cross-Subjects

**Figure 5:**
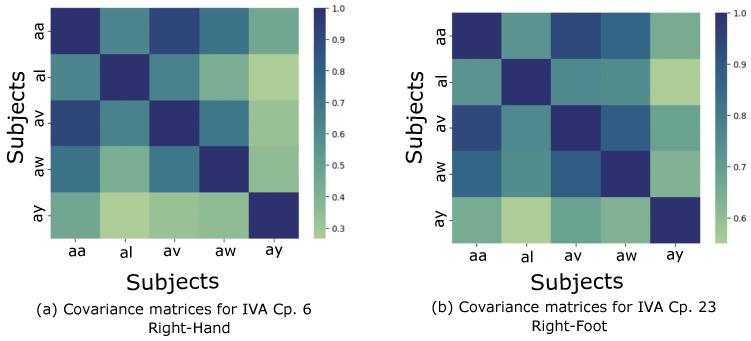
Examples of SCV covariance matrices obtained through IVA for the DS4a considering right hand and right foot movements.

In Section 2.1, we mentioned the SCVs and how they are extracted through IVA. In this section, we present the relation between the results achieved from SCV covariance matrices, obtained through the use of the estimated sources $y_{m}^{\left[k\right]}$, and the DS4a cross-subjects. Figure 5 shows two SCV covariance matrix examples extracted from IVA components (IVA Cp.). These results are based on the use of 37 channels, which achieved the best outcome in the previous analysis. In Figure 5a, we present the covariance matrix obtained from the SCVs for the right hand movement and IVA component 6 as an example. As can be seen, two cases present a higher cross-correlation: “aa” with “av”, which achieves a value of 0.909; and “aa” with “aw” which achieves 0.708. The second example is based on the right foot movement and IVA component 23, shown in Figure 5b, where the highest correlation of 0.936 was achieved also among subjects “aa” and “av”, but other relevant correlations were reached between subjects “av” and “aw”, with a value of 0.887, and subjects “al” and “aw” with a value of 0.749.

Based on the covariance measure obtained from the SCVs, Table 1 and Table 2 show the results obtained for the five highest correlations cross-subjects, for each MI class (the same considered in the discussion above), IVA component, and subject. For this reason, the same IVA component or subject may appear more than once, meaning that it contributed again to one of the highest correlation situations. The correlation values shown were computed based on an average of 10-fold.

In Section 4.2, we computed the KDE and investigated the IVA initialization for the five subjects, having noted a similar distribution between subjects “aa” and “av”, and subjects “aw” and “ay” in Figure 3. Comparing these results with the ones derived from Table 1 and Table 2, we can observe an analogous behavior, i.e., subjects “aa” and “av” present a high cross-correlation when considering IVA components 9 and 13, for right hand movement, and components 24 and 3 for right foot MI. Additionally, for subject “av”, four of the five selected correlations were related to subject “aa” in Table 2, which represents a strong relation between them. In the second case, for subjects “aw” and “ay”, higher correlations emerged from IVA components 9 and 2 for the right hand and right foot, respectively. These results present a significant correlation across subjects (around 0.45) and an intriguing perspective, since the KDE distribution of the subjects could lead to a potential clustering of MI-based BCI patients, from which similar features could be exploited to improve classification performance or aid the development of a global model.

### 5.4. Deep Learning Approaches

Thus far, we have combined IVA feature extraction with two different classification algorithms. IVAS presented the best performance and provided valuable features using, as parameters, q=4 and μ=1. In order to explore the deep learning approach and evaluate the influence of the components extracted from IVA, the obtained independent components were applied to the EEGNet model (IVAEN) and EEG-Inception model (IVAEI). It is important to note that the AR step was not applied in this case. These methods were implemented and trained using the Braindecode library [41]. Moreover, we applied an augmentation data technique based on Gaussian white noise and/or replication [42]. Having chosen the parameters for each algorithm, Table 3 presents the final results for the DS4a dataset.

The second-best result for subjects “aa” and “al” was obtained using IVAEN. IVAEI achieved 92.1% and 91.4% for subjects “aw” and “ay”, respectively. The latter matched the WPD method’s performance, while the former showed a slight difference of 3.3%. The IVAS algorithm was able to find the third-best result for subject “av” (the most difficult subject to be classified) when compared to other results in the literature. On average, it is possible to note that the IVAEI results showed the second-best average accuracy performance of 86.7% and a standard deviation of 9.4.

## 6. Conclusions and Future Perspectives

In this work, we have presented a feature extraction method for motor imagery classification through EEG signals. This approach minimizes the mutual information to achieve independent vector analysis through multiple datasets. The proposed method was evaluated using the BCI Competition III Dataset 4a with five subjects. Although there are some limitations in terms of tested datasets, when we concentrated on a single well-established one, we were able to conduct a more in-depth and focused analysis, using this dataset for a proof of concept. For DS4a, the IVAEI algorithm obtained the best results, reaching an accuracy of 86.7%, considering the average between all subjects. Moreover, we showed how the selection of the algorithm parameters such as step sizes, number of AR coefficients, and number of EEG channels affect the algorithm’s performance, and how the components’ correlations identified from IVA could lead to another interesting result, concerning the clustering of MI-based BCI patients. In the future, we consider investigating a generalization of the method, extending the work using more complex datasets, exploring the clustering problem, or even integrating multimodal data for enhanced feature extraction. Furthermore, IVA can be incorporated into a number of remaining challenging tasks in the EEG context, such as online analysis, transfer learning, and feedback systems. While IVA has been traditionally used as an offline method, new extensions allow for the regression of previous results using a new subject’s data without the need to perform a complete decomposition [43,44], which would allow for the required flexibility. A possible transfer learning approach is another interesting concept that can be exploited, considering that the correlation is naturally incorporated into the process to enhance the model’s performance and robustness.

Concerning the deep learning approaches, we focused on comparing different feature extraction methods where deep learning algorithms were used only for the final classification stage, since the feature extraction stage plays an important role in the classification of EEG signals for BCI systems. Additionally, the comparison between methods with and without feature extraction could yield insightful results regarding the use of IVA to obtain network weights, something that was not on the scope of this paper. In this sense, IVA might be better suited for a separate stage, but incorporating a feedback system could be a promising approach for future developments.

Finally, despite IVA’s strong identifiability properties, the direct interpretation of sensor domain components remains challenging. Future research could enhance interpretability by transforming data to the spectral domain or using features like event-related potentials (ERPs), as proposed in previous studies [21,45,46].

## Figures and Tables

**Table 1:** Main IVA component correlations per subject for right hand (DS4a) and the similarities between subjects compared with the KDE analysis.

Right Hand						
	IVA Cp.	Cp. 9	Cp. 21	Cp. 18	Cp. 13	Cp. 17
“aa”	Cross-Subj.	“av”	“al”	“ay”	“av”	“ay”
	Correlation	0.493	0.463	0.450	0.435	0.432
	IVA Cp.	Cp. 21	Cp. 30	Cp. 21	Cp. 8	Cp. 23
“al”	Cross-Subj.	“aa”	“av”	“av”	“aa”	“aa”
	Correlation	0.463	0.458	0.431	0.430	0.422
	IVA Cp.	Cp. 9	Cp. 30	Cp. 13	Cp. 9	Cp. 21
“av”	Cross-Subj.	“aa”	“al”	“aa”	“ay”	“al”
	Correlation	0.493	0.458	0.435	0.434	0.431
	IVA Cp.	Cp. 9	Cp. 30	Cp. 23	Cp. 26	Cp. 23
“aw”	Cross-Subj.	“ay”	“aa”	“al”	“aa”	“aa”
	Correlation	0.418	0.418	0.414	0.407	0.398
	IVA Cp.	Cp. 18	Cp. 9	Cp. 17	Cp. 2	Cp. 9
“ay”	Cross-Subj.	“aa”	“av”	“aa”	“aa”	“aw”
	Correlation	0.450	0.434	0.432	0.429	0.418

**Table 2:** Main IVA component correlations per subject for right foot (DS4a) and the similarities between subjects compared with the KDE analysis.

Right Foot						
	IVA Cp.	Cp. 16	Cp. 2	Cp. 24	Cp. 6	Cp. 3
“aa”	Cross-Subj.	“al”	“al”	“av”	“ay”	“av”
	Correlation	0.535	0.470	0.465	0.450	0.444
	IVA Cp.	Cp. 16	Cp. 16	Cp. 2	Cp. 2	Cp. 16
“al”	Cross-Subj.	“aw”	“aa”	“aa”	“ay”	“av”
	Correlation	0.540	0.535	0.470	0.463	0.448
	IVA Cp.	Cp. 24	Cp. 16	Cp. 3	Cp. 11	Cp. 8
“av”	Cross-Subj.	“aa”	“al”	“aa”	“aa”	“aa”
	Correlation	0.465	0.448	0.444	0.437	0.426
	IVA Cp.	Cp. 16	Cp. 2	Cp. 16	Cp. 16	Cp. 11
“aw”	Cross-Subj.	“al”	“ay”	“av”	“aa”	“al”
	Correlation	0.540	0.449	0.420	0.420	0.410
	IVA Cp.	Cp. 2	Cp. 6	Cp. 2	Cp. 7	Cp. 27
“ay”	Cross-Subj.	“al”	“aa”	“aw”	“aa”	“aa”
	Correlation	0.463	0.450	0.449	0.438	0.436

**Table 3:** Accuracy and standard deviation obtained in classifying BCI Competition III Dataset 4a.

	Subjects					
**Methods**	**“aa”**	**“al”**	**“av”**	**“aw”**	**“ay”**	**Average ± Sd**
SCS-NMF	64.2	92.67	60.0	72.6	55.3	68.9 ± 14.7
DPL	81.5	100	60.2	83.0	79.4	80.8 ± 14.1
BECSP	77.7	**100**	73.9	84.8	88.1	84.9 ± 10.1
WPD	**96**	92.3	**88.9**	**95.4**	**91.4**	**92.8** ± **2.9**
IVAS	71.8	96.1	**70.0**	84.3	85.0	81.4 ± 10.7
IVAK	59.6	93.6	61.1	72.1	68.6	71.0 ± 12.2
IVAEN	**87.8**	**98.5**	66.4	68.6	82.6	80.8 ± 13.4
**IVAEI**	84.3	96.4	69.3	**92.1**	**91.4**	**86.7** ± **9.4**

## Data Availability

Data are contained within the article.

## Abbreviations

The following abbreviations are used in this manuscript:

AR	Autoregressive
BCI	Brain–computer interface
BSS	Blind source separation
DS4a	BCI Competition III Dataset 4a
EEG	Electroencephalogram
ICA	Independent component analysis
IVA	Independent vector analysis
JBSS	Joint blind source separation
KDE	Kernel density estimation
KNN	K-nearest neighbors
MI	Motor imagery
SCV	Source component vectors
SVM	Support vector machines
